# Nicotine pharmacokinetics and subjective responses after using nicotine pouches with different nicotine levels compared to combustible cigarettes and moist smokeless tobacco in adult tobacco users

**DOI:** 10.1007/s00213-022-06172-y

**Published:** 2022-07-23

**Authors:** Jianmin Liu, Jesse Rensch, Jingzhu Wang, Xiaohong Jin, Andrea Vansickel, Jeffery Edmiston, Mohamadi Sarkar

**Affiliations:** Center for Research and Technology, Altria Client Services LLC, 601 East Jackson Street, Richmond, VA 23219 USA

**Keywords:** Novel oral tobacco products, Oral tobacco–derived nicotine products, Nicotine pouches, Abuse potential, Dependence potential, Urges to smoke, Subjective measures, Nicotine pharmacokinetics

## Abstract

**Rationale:**

Oral tobacco–derived nicotine products include on!® nicotine pouches (NPs) which are tobacco-leaf free and available in multiple flavors and nicotine levels. Switching completely to NPs from cigarettes and moist smokeless tobacco (MST) has the potential to reduce harm for adult tobacco consumers. However, the dependence potential of NPs is not established. Therefore, we characterized the abuse potential of NPs with different nicotine levels compared to cigarettes and MST.

**Objectives:**

To evaluate nicotine pharmacokinetics (PK) and subjective effects of NPs (ranging from 1.5 to 8 mg nicotine) compared to own brand cigarettes (OBCs) and MST (OBMST).

**Methods:**

We used a randomized, in-clinic, partial single-blind, 7-way crossover design to assess nicotine PK and subjective effects in dual users of cigarettes and MST.

**Results:**

The mean nicotine *C*_max_ for NPs increased with nicotine level, ranging from 3.5 ng/mL (1.5 mg NP) to 15.4 ng/mL (8 mg NP), compared with 12.2 ng/mL for OBCs and 9.8 ng/mL for OBMST. Nicotine *t*_max_ was much longer for all NPs and OBMST (32.5–34.4 min) compared to OBCs (8.5 min). Reductions in urges to smoke after use of the 2 mg, 3.5 mg, and 8 mg NPs were not statistically different (*p* > 0.05) relative to OBC. Also, NPs resulted in lower ratings of positive subjective effects relative to OBCs and OBMST.

**Conclusions:**

Overall, based on the study results and literature reported nicotine PK values for cigarettes and MST, the abuse potential of NPs is not likely to be higher than OBCs and OBMST. NPs may be potentially acceptable switching products for users of cigarettes and MST products.

**Supplementary Information:**

The online version contains supplementary material available at 10.1007/s00213-022-06172-y.

## Introduction

Cigarette smoking remains the leading cause of preventable premature death and disease in the USA. Smoking-related diseases are caused by harmful and potentially harmful constituents (HPHCs) and other compounds in cigarettes that are inhaled in the smoke (US Department of Health and Human Services 2014). Many in public health (Gottlieb and Zeller 2017; Hatsukami et al. 2007; Zeller et al. 2009) have acknowledged that a continuum of risk exists among tobacco products, with conventional, combustible cigarettes at the higher end and non-combustible products on the lower end. In recent years, there has been a rapid growth of a variety of innovative, non-combustible products, including oral tobacco–derived nicotine (OTDN) products. Nicotine pouches (NPs) are one example of OTDN products that contain pharmaceutical grade nicotine derived from tobacco, flavors, and other ingredients used in foods. NPs are tobacco-leaf free and contain far fewer HPHCs than cigarette smoke (Wagner et al. 2020). Switching to NPs, therefore, presents a potential harm reduction opportunity among the ~ 34.1 million (Cornelius et al. 2020) adult cigarette smokers (AS) and ~ 5.9 million (Cornelius et al. 2020) adult moist smokeless tobacco (MST) users, particularly those that are unable or unwilling to quit tobacco products.

To date, there are only two published studies that inform the abuse potential of NPs (Lunell et al. 2020; Rensch et al. 2021). Lunell et al. (2020) compared Zyn® NPs containing 3 and 6 mg nicotine with 8 mg General snus, and Zyn® NPs containing 8 mg nicotine with 18 mg Longhorn moist snuff in a study of snus users. The authors observed that the two higher levels of Zyn® (6 and 8 mg) delivered nicotine as quickly and to a similar extent as the comparator MST. The authors had also measured subjective effects (“head buzz”) and observed no obvious correlation between nicotine levels and the maximum score for “head buzz.” Rensch et al. recently reported nicotine pharmacokinetics (PK) and subjective effects of six flavors of on!® NPs containing 4 mg nicotine in AS compared to own brand cigarettes (2021). This study was specifically designed to assess abuse potential of the NPs investigated relative to cigarettes. The authors concluded that based on the PK profiles and subjective responses, the NPs were likely to be associated with lower abuse potential than cigarettes. The findings from the study also indicated that flavor does not appear to influence nicotine PK or subjective responses, and the NPs may be potentially acceptable switching products for AS and adult MST users (Rensch et al. 2021). We present here results from a randomized, controlled, clinical study with varying nicotine levels to further inform the abuse potential of the on!® NPs. The rationale for this study is to add to the limited scientific knowledge regarding the abuse potential of NP products.

This study evaluated the nicotine PK and subjective effects of five mint-flavored NPs with different nicotine levels relative to participants’ own brand cigarettes (OBCs) and own brand MST (OBMST) in adult dual users of cigarettes and MST. Evaluation of nicotine PK, including the time-course and amount of nicotine delivery, is used to assess tobacco products’ abuse potential; whereby, tobacco products with a greater rate and extent of nicotine delivery are more likely to be used repeatedly (Henningfield and Keenan 1993). The subjective responses to tobacco products are measured using well-established questionnaires, which can serve as proxy measures of the positive, rewarding effects as well as the negative, adverse effects of tobacco products that may influence subsequent use behavior and inform abuse potential (Carter et al. 2009; Cobb et al. 2010; Cox et al. 2001; Gray et al. 2008; Hanson et al. 2009; Vansickel et al. 2010). The purpose of this study was to assess the nicotine PK profiles and subjective measure responses from use of NPs which will inform the abuse potential of the NPs relative to adult smokers’ OBCs and smokeless tobacco (ST) users’ OBMST. We hypothesize that the abuse potential of the NPs tested will not be higher than OBCs and OBMST products.

## Materials and methods

### Participants

Participants were recruited and screened at three different study sites: Celerion (Lincoln, NE), Bio-Kinetic Clinical Applications, LLC (Springfield, MO), and Midwest Clinical Research Center, LLC (Dayton, OH). The recruitment utilized the database of the potential study population of AS and MST users at each site, as well as social media videos, radio, print, and digital advertising. Healthy adult dual users (smokers who also used MST) age 21 to 65 years who fulfilled all inclusion criteria and met none of the exclusion criteria were eligible to participate. The eligible participants checked in to the clinic at Celerion in Lincoln, NE, where the study was conducted from September to November in 2019. Participants were self-affirmed dual users of cigarettes (consumption of at least 10 cigarettes per day [CPD]) and MST (consumption of at least one can per week) for at least 12 months and had urine cotinine levels ≥ 500 ng/mL at screening. Potential participants were excluded if they had used any OTDN pouch products within 30 days prior to the screening visit or reported any plans or attempts to quit smoking or using MST in the past 3 months.

### Study products

The test products were mint-flavored on!® NPs, which contain tobacco-derived nicotine bitartrate dihydrate at five different nicotine levels (1.5 mg, 2 mg, 3.5 mg, 4 mg, and 8 mg), microcrystalline cellulose, sodium carbonate, flavoring ingredients, and binders within a permeable, non-dissolving pouch. Participants’ OBCs and OBMST were used as the reference products. The products investigated in the study are commercially available products. We note that the 1.5 mg and 3.5 mg test products described in this manuscript were identified as 1 mg and 3 mg products, respectively, in the study documents.

### Study design

This study used a randomized, partial single-blind, 7-way crossover design. Participants who passed screening completed an ambulatory 5-day (consecutive or non-consecutive) product trial (Stage 1), in which they were provided with one can (20 pouches) of each of the five nicotine levels of the NPs. During this product trial period, participants were instructed to use at least one NP of each nicotine level, starting from the lowest on the first day and moving to the higher levels sequentially in the subsequent product trial days, concluding with use of at least one 8 mg NP for at least 30 min to confirm tolerability. Product use behavior (e.g., the number and nicotine level of NPs used per day, the number used each time, and the number of days used) was documented in a diary by participants.

**Fig. 1:**
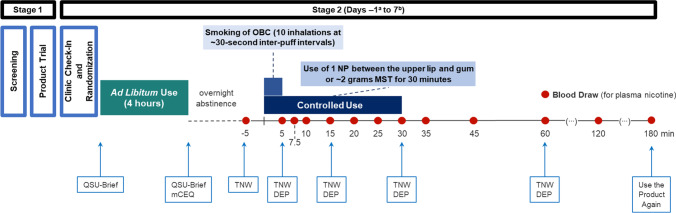
Study design. ^a^There was no controlled product use period on day − 1; participants completed the ad libitum use period followed by overnight abstinence on day − 1. The participants stayed in the clinic for a total of 8 days (day − 1 through day 7). ^b^There was no afternoon ad libitum use period after the controlled use period in the morning on the last study day, day 7. DEP, Direct Effects of Product Questionnaire; mCEQ, Modified Cigarette Evaluation Questionnaire; MST, moist smokeless tobacco; NP, nicotine pouch; OBC, own brand cigarette; QSU, Questionnaire on Smoking Urges; TNW, Tobacco/Nicotine Withdrawal Questionnaire

Following the product trial, eligible participants checked in to the clinic and were randomized to one of seven product sequences and were only allowed to use assigned study products at scheduled times (Stage 2). During the 8-day, in-clinic product use, participants knew whether the study product was an NP, OBC, or OBMST, but were blinded to the nicotine level of the NP (partial single-blind). The randomized, 7-way, crossover design allowed each participant to use all five nicotine levels and their OBCs and OBMST products in this within-subject design. Participants used their assigned product ad libitum for 4-h periods approximately 15 h before the controlled product use period. The 15-h overnight abstinence from use of any tobacco- or nicotine-containing products was monitored by the clinic staff. Participants completed subjective measure questionnaires at pre-determined timepoints (see Supplementary Table 1). During the controlled product use periods, participants used the assigned product under the following conditions: smoked one cigarette with 10 inhalations at ~ 30-s inter-puff intervals, used one NP by placing the pouch between the upper lip and gum for 30 min, or used ~ 2 g (± 0.01 g) of OBMST for 30 min. The 30-min use of the NP reflects an “extreme” condition of use (the marketed product indicates “Enjoy for up to 20 min” on the packaging). Participants completed the controlled use subjective measures questionnaires at pre-determined timepoints (see Fig. 1 and Supplementary Table 1).

Over each 4-h ad libitum product use period, clinic staff documented the start and stop time for each product use, the total number of NPs or cigarettes used, the number of NPs used each time, the number of OBMST quids (a quid refers to a pinch of OBMST that a participant placed in the mouth at the time of use) used, the amount of time in the mouth during each NP or OBMST use, and the total weight of OBMST used, as applicable.

### Nicotine pharmacokinetics

Plasma nicotine concentrations were measured from blood samples collected at ~ 5 min prior to and at 5, 7.5, 10, 15, 20, 25, 30, 35, 45, 60, 120, and 180 min following the start of each controlled product use period. PK parameters (including area under the curve [AUC_(0–180)_], maximum nicotine concentration [*C*_max_], time of the maximum measured plasma concentration [*t*_max_], apparent first-order terminal elimination rate constant [kel], and apparent first-order elimination half-life [*t*_½_]) were calculated from the baseline-adjusted plasma nicotine concentration–time data using Phoenix® WinNonlin® version 7.0.

### Subjective measures

A summary of the items contained in and administration timing of the previously published subjective measures questionnaires completed during the ad libitum and controlled product use periods is provided in Supplementary Table 1. Participants completed the subjective measures questionnaires using a tablet with preloaded sequences programed by Clinical Ink (Horsham, PA). During ad libitum in-clinic use, the Questionnaire on Smoking Urges-Brief (QSU-Brief) was administered before and after each product use period to assess desire and intention to smoke and anticipation of relief from negative affects (Cox et al. 2001). The Modified Cigarette Evaluation Questionnaire (mCEQ; further modified for use with NPs and OBMST) was completed after each ad libitum product use period to assess satisfaction, enjoyment of sensations, psychological reward, craving, and aversion (Rose et al. 2010; St. Helen et al. 2016).

To assess the magnitude, onset, and offset of product reinforcing effects during controlled product use, an in-the-moment response to a visual analog scale (VAS) of the Tobacco/Nicotine Withdrawal (TNW) Questionnaire was collected before, during, and after each use period and response to the Direct Effects of Product (DEP) Questionnaire was captured during and after each controlled use period. Evidence from Hanson et al. (2009) indicates that the items included in the TNW and DEP Questionnaires are sensitive enough to detect differences between tobacco products. As a measure of the overall likelihood of subsequent use behavior, response to the Use the Product Again (adapted from Griffiths et al. 2003) bipolar VAS, anchored with “definitely would” and “definitely would not” at either end and “don’t care” in the middle, was also assessed after each controlled use period.

### Safety assessments

Clinical safety evaluations included clinical laboratory testing (serum chemistry, hematology, and urinalysis), drug and alcohol screens, pregnancy tests (females), confirmation of tobacco use, physical examinations, and electrocardiograms. Vital signs (blood pressure, pulse/respiration rates, and body temperature) were measured at screening, check in, and at the end of the study. Adverse events (AEs) were monitored and reported from the first NP use until the end of study.

### Statistical analysis

The primary outcome variables were nicotine PK parameters (*C*_max_ and AUC_(0–180)_), maximum reduction in response, under controlled use conditions, relative to pre-use for TNW items (*E*_max__TNW) and the maximum response on the DEP items following the product use under controlled use conditions (*E*_max__DEP). The hypothesis, based on the primary outcome variables, was that the *C*_max_, *E*_max__TNW, and *E*_max__DEP following the controlled use of NPs tested are not statistically different (*α* level of 0.05) from that observed for OBCs or OBMST. Data were analyzed using the statistical methods described in a previous publication (Rensch et al. 2021). SAS software (version 9.4, Cary, NC) was used for all data presentation and summarization including statistical analyses, summary tables, graphs, and data listings. A linear mixed model for analysis of variance was performed on the natural log-transformed AUC and *C*_max_. The model included sequence, study product, and period as fixed effects and subject-nested-within-sequence as a random effect.

### Sample size estimation

Based on a literature search, typical sample sizes range from 10 to 32 participants for studies examining the PK and subjective effects across different tobacco/nicotine conditions (Carter et al. 2009; Cobb et al. 2010; Cox et al. 2001; Gray et al. 2008; Hatsukami et al. 2004; Kotlyar et al. 2007; Lunell and Curvall 2011; Perkins et al. 1997). The sample size of 30 participants was considered adequate for the current study design.

## Results

Out of 66 people screened for this study, 36 people failed screening procedures. A total of 30 participants (29 males and 1 female) were enrolled, completed the 5-day ambulatory product trial, and checked in to the clinic on day − 1. All 30 participants were randomized to one of seven study product sequences, and 28 participants completed the study. One male participant chose to withdraw from the study on day 2 due to unrelated AEs (body aches, chills, and fatigue), and one male participant withdrew on day 5 due to a family emergency.

The study population was predominantly white (90%) and male (97%) with an average age of 35 years (Table 1). All participants were cigarette smokers and concurrent MST users at screening, had smoked an average of about 15 CPD for 14 years, and used ~ 3.5 cans of MST per week for 12 years. Two-thirds of study participants (67%) reported using non-menthol cigarettes and the majority (97%) reported using long-cut MST.

**Table 1:** Demographics and product use history

Parameter	*N* = 30
Sex, *n* (%)	
Female Male	1 (3) 29 (97)
Race, *n* (%)	
White Black	27 (90) 3 (10)
Ethnicity, *n* (%)	
Hispanic or Latino Not Hispanic or Latino	1 (3) 29 (97)
Age, years	34.9 (9.63)
BMI, kg/m^2^	28.5 (5.08)
Number of cigarettes smoked per day	15.3 (4.12)
Number of years of smoking	14.3 (9.49)
Number of cans of MST product used per week	3.5 (2.28)
Number of years of MST product use	11.9 (8.31)

Data are presented as mean (standard deviation) unless otherwise noted

*BMI* body mass index, *MST* moist smokeless tobacco

During the product trial period, participants reported using about five NPs per day. Overall, during each 4-h ad libitum product use period, the average pouch consumption ranged from ~ 6 pouches (8 mg) to ~ 18 pouches (1.5 mg). The mean cigarette consumption was ~ 10 cigarettes, and OBMST use was ~ 3 quids/pinches. The average length of time that the NPs were used in the mouth ranged from ~ 29 (1.5 mg) to ~ 56 (2 mg) min. OBMST quids were used for ~ 42 min per use during the 4-h ad libitum product use period.

### Nicotine pharmacokinetics

Plasma nicotine PK parameters were baseline-adjusted (Supplementary Table 2) because measurable nicotine levels (≥ 0.2 ng/mL) were observed at baseline prior to product use under controlled conditions, in most study participants (Supplementary Table 2). The *t*_max_ of nicotine uptake from all five NPs (range: 32.5 to 33.9 min) was slower than OBCs (8.5 min) and was similar to OBMST (34.4 min; Supplementary Table 2). The shape of the nicotine PK profiles was similar among all the NPs and OBMST products. The geometric least squares mean *C*_max_ and AUC_(0–180)_ values increased with the increasing nicotine level of the NPs (Table 2).

**Table 2:** Summary of statistical comparisons of baseline-adjusted plasma nicotine pharmacokinetic parameters

NP nicotine level or OBMST	LS mean *C*_max_, ng/mL and AUC, ng min/mL (*n*)	Comparison with OBCs		Comparison with OBMST	
		Geometric LS mean ratio (test/Ref^a^), % (95% CI)	*p*-value	Geometric LS mean ratio (test/OBMST), % (95% CI)	*p*-value
1.5 mg NP					
*C*_max_ AUC	3.2 (30) 306.0 (29)	30.8 (25.5, 37.1) 38.1 (31.7, 45.8)	< 0.0001 < 0.0001	35.4 (29.3, 42.7) 31.0 (25.8, 37.2)	< 0.0001 < 0.0001
2 mg NP					
*C*_max_ AUC	4.6 (29) 426.6 (29)	43.7 (36.2, 52.8) 53.1 (44.2, 63.8)	< 0.0001 < 0.0001	50.3 (41.6, 60.7) 43.2 (36.0, 51.9)	< 0.0001 < 0.0001
3.5 mg NP					
*C*_max_ AUC	7.1 (28) 699.0 (28)	67.0 (55.4, 81.1) 87.0 (72.3, 104.7)	< 0.0001 0.1405	77.1 (63.7, 93.3) 70.8 (58.8, 85.2)	0.0077 0.0003
4 mg NP					
*C*_max_ AUC	8.4 (28) 796.0 (28)	80.1 (66.2, 96.9) 99.1 (82.4, 119.3)	0.0227 0.9251	92.1 (76.1, 111.4) 80.6 (67.0, 97.0)	0.3925 0.0226
8 mg NP					
*C*_max_ AUC	14.5 (28) 1441 (27)	137.4 (113.5, 166.2) 179.4 (148.8, 216.3)	0.0013 < 0.0001	157.9 (130.5, 191.1) 145.9 (121.0, 175.9)	< 0.0001 0.0001
OBMST					
*C*_max_ AUC	9.2 (29) 987.7 (29)	87.0 (72.0, 105.1) 123.0 (102.4, 147.7)	0.1465 0.0271	— —	— —

*AUC* area under the nicotine concentration–time curve from time 0 to 180 min, *CI* confidence interval, *C*_*max*_ maximum measured plasma concentration, *LS* least squares, *NP* nicotine pouch, *OBC* own brand cigarette, *OBMST* own brand moist smokeless tobacco

^a^Reference (OBC): *C*_max_ = 10.5 ng/mL; AUC = 803.1 ng min/mL; *n* = 29

The *C*_max_ geometric mean values ranged from 3.2 ng/mL (1.5 mg NP) to 14.5 ng/mL (8 mg NP). The AUC_(0–180)_ ranged from 306 ng*min/mL (1.5 mg NP) to 1441 ng*min/mL (8 mg NP). The 1.5, 2, 3.5, and 4 mg NPs resulted in statistically significantly lower (*p* < 0.05) *C*_max_ values, while the 8 mg NP resulted in a statistically significantly (*p* < 0.05) higher *C*_max_ relative to participants’ OBCs. The AUC_(0–180)_ values were statistically significantly lower (*p* < 0.0001) for 1.5 mg and 2 mg NPs relative to participants’ OBC AUC_(0–180)_. No statistically significant differences were observed for AUC_(0–180)_ for the 3.5 and 4 mg NPs, and the 8 mg NP’s AUC_(0–180)_ was significantly higher (*p* < 0.0001) relative to participants’ OBC.

Relative to participants’ OBMST, the *C*_max_ for the 1.5, 2, and 3.5 mg NPs was significantly lower (*p* < 0.0001). The 4 mg NP resulted in similar *C*_max_, and the 8 mg NP resulted in significantly higher *C*_max_ (*p* < 0.0001) relative to participants’ OBMST. The 1.5, 2, 3.5, and 4 mg NPs resulted in significantly lower (*p* < 0.05 for all comparisons) AUC_(0–180)_, while the 8 mg NP resulted in a significantly higher (*p* < 0.0001) AUC relative to participants’ OBMST. No indications of non-linear pharmacokinetics were observed based on the estimated nicotine elimination half-life values at the nicotine levels examined.

### Subjective responses

While there was a reduction in the QSU-Brief factor scores (Factor 1 — desire and intention to smoke and Factor 2 — anticipation of relief from negative affect) after 4-h ad libitum use of the NPs, the magnitude of reduction relative to baseline was much smaller than with participants’ OBCs. Additionally, no association was apparent between the nicotine levels and the factor scores. The mean Factor 1 scores ranged from 5.9 to 6.5 before the ad libitum use period; scores decreased to a range of 4.8 to 5.2 for NPs, and 2.5 for OBCs, and 4.2 for OBMST at the end of the ad libitum use period. Similarly, mean Factor 2 scores reduced after use from 3.9 to 4.4 at baseline to a range of 2.8 to 3.0 for NPs, and 2.0 and 2.5 for OBCs and OBMST, respectively.

The mean factor scores for satisfaction, psychological reward, enjoyment of sensation, and craving reduction from the mCEQ after 4-h ad libitum use were generally similar with the use of NPs among all nicotine levels and were lower than mean scores for participants’ OBCs and OBMST (Supplementary Fig. 1). The aversion factor scores (average values ranging from 1.2 to 1.5) were similar between the 1.5, 2, 3.5, and 4 mg NPs, OBCs, and OBMST. The 8 mg NP exhibited the highest aversion score (average 2.3).

Based on responses to the TNW Questionnaire under controlled use conditions, the magnitude of the maximum reduction in urges to smoke was significantly larger for OBC relative to the 1.5 and 4 mg NPs (*p* < 0.05). The magnitude of the reduction in urges to smoke was not statistically significantly different between the OBC and after use of the other NPs (2, 3.5, and 8 mg; *p* > 0.05; Table 3 and Supplementary Table 3). The magnitude of reduction in craving a cigarette was statistically significantly lower (*p* < 0.05) for the 1.5, 2, 3.5, and 4 mg NPs and not statistically different for the 8 mg NP compared to OBCs. Compared with OBMST, responses to craving a cigarette on the TNW Questionnaire were significantly lower for the 1.5, 2, 3.5, and 4 mg NPs and responses to urges to smoke were significantly lower for the 1.5 and 4 mg NPs (*p* < 0.05).

**Table 3:** Mean (SD) and median *E*_max_ VAS subjective ratings of the of NPs, OBCs, and OBMST

		1.5 mg NP	2 mg NP	3.5 mg NP	4 mg NP	8 mg NP	OBC	OBMST
Tobacco/Nicotine Withdrawal Questionnaire								
Urges to smoke	Mean (SD) Median	17.7 (20.1) 12.0	22.6 (22.4) 18.0	23.1 (28.1) 18.0	18.8 (24.4) 15.5	27.1 (28.2) 17.5	29.9 (26.2) 22.0	29.6 (31.8) 17.0
Craving a cigarette	Mean (SD) Median	19.3 (17.7) 17.0	19.6 (21.0) 17.0	19.8 (26.8) 14.0	21.6 (24.2) 19.0	28.0 (26.7) 22.5	30.9 (26.7) 25.0	32.8 (29.1) 27.0
Direct Effects of Product Questionnaire								
Is the product “Pleasant” right now?	Mean (SD) Median	48.0 (24.1) 51.0	54.1 (21.6) 57.0	53.3 (21.4) 52.5	52.1 (23.9) 52.5	51.3 (24.5) 50.5	69.3 (28.3) 77.0	65.7 (22.7) 68.0
Is the product “Satisfying” right now?	Mean (SD) Median	46.3 (23.4) 46.5	53.7 (21.5) 58.0	53.2 (22.6) 52.5	50.8 (22.8) 55.5	50.9 (25.3) 55.5	70.8 (26.2) 75.0	67.0 (24.1) 67.0
Is the product making you feel “Calm” right now?	Mean (SD) Median	41.8 (23.9) 41.5	49.0 (21.9) 49.0	47.8 (22.8) 51.5	43.2 (27.6) 47.0	46.9 (28.6) 48.5	63.6 (28.4) 65.0	62.0 (29.5) 74.0
Is the product helping you “Concentrate” right now?	Mean (SD) Median	34.9 (23.6) 33.5	43.9 (25.4) 50.0	42.0 (23.9) 45.5	38.8 (25.9) 42.5	39.1 (27.6) 45.0	49.9 (29.5) 56.0	49.3 (26.6) 47.0
Is the product making you feel more “Awake” right now?	Mean (SD) Median	40.7 (27.9) 33.0	44.0 (25.3) 53.0	44.4 (25.8) 50.0	37.5 (26.3) 41.5	40.4 (26.9) 46.0	49.6 (30.1) 52.0	50.9 (28.9) 51.0
Is the product making you feel “Sick” right now?	Mean (SD) Median	16.7 (18.5) 7.0	14.3 (18.2) 5.0	14.7 (18.2) 7.5	18.0 (20.4) 11.0	26.2 (30.1) 11.5	21.2 (26.0) 10.0	20.0 (27.5) 6.0
Is the product reducing your “Hunger” for food right now?	Mean (SD) Median	25.4 (24.0) 21.5	27.9 (24.7) 28.0	27.8 (27.3) 19.0	29.4 (25.2) 28.0	32.9 (28.0) 30.5	38.1 (30.6) 38.0	36.6 (31.4) 44.0
Would you like “More” of the product right now?	Mean (SD) Median	65.2 (28.0) 69.0	65.4 (29.6) 70.0	63.2 (26.7) 67.0	61.7 (27.7) 61.5	51.0 (31.8) 51.5	73.1 (22.9) 74.0	67.5 (27.5) 68.0

The Tobacco/Nicotine Withdrawal (TNW) Questionnaire and the Direct Effects of Product (DEP) Questionnaire are described in Supplementary Table 1. *E*_max_ represents the maximum reduction from pre-use in the score on the TNW assessment; *E*_max_ represents the maximum score on the DEP assessment

*NP* nicotine pouch, *OBC* own brand cigarette, *OBMST* own brand moist smokeless tobacco, *SD* standard deviation, *VAS* visual analog scale

**Fig. 2:**
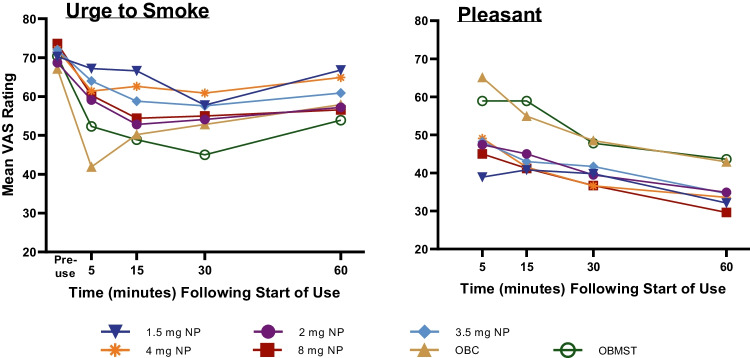
Mean visual analog scale scores over time for subjective ratings before and/or during controlled use. Items assessed in the Tobacco/Nicotine Withdrawal and the Direct Effects of Product Questionnaires are described in Supplementary Table 1. NP, nicotine pouch; OBC, own brand cigarette; OBMST, own brand moist smokeless tobacco; VAS, visual analog scale

Subjective ratings for the NPs on the DEP Questionnaire were either statistically significantly lower (*p* < 0.05) than or similar to participants’ OBCs (Table 3; Supplementary Table 3. More participants responded positively to the Use the Product Again Questionnaire for OBCs (77%) or OBMST (93%) than they did for NPs (range: 46% [8 mg] to 66% [1.5 mg]; Supplementary Fig. 2). Among the NPs tested, the 8 mg NP engendered the lowest percentage of positive (46%) responses, the highest percentage of negative (36%) and neutral (18%) responses, relative to OBC and OBMST. The time-course of changes in “urges to smoke” suggests that the maximum reduction during use of the NPs occurred later than for OBCs (15 to 30 min vs. 5 min) and at a similar time to OBMST (30 min; Fig. 2).

### Adverse event reports

No serious adverse events (AEs) were reported, and no participants were discontinued due to AEs. After study product randomization on day − 1, 61 AEs were reported by 20 participants (67%), with 59 of the events being mild in severity and two events (headache in the OBC group and nausea in the 8 mg NP group) being moderate in severity. Headache was the most frequently reported event, experienced by eight participants (27%), followed by nausea, experienced by six participants (20%). All remaining events were reported by four or fewer participants (≤ 13%) each. Seven events were considered to be likely related to study products and 13 events possibly related. The likely/possibly related events occurred across study products and included, but were not limited to, nausea, vomiting, dizziness, and headache, and were as typically expected with use of oral nicotine products.

## Discussion

Results of this partial single-blind, randomized, 7-way crossover study suggest that, among current AS and MST users, the abuse potential of NPs tested is not likely to be higher than cigarette or MST. The study results and literature reported PK values for cigarettes (D’Ruiz et al. 2015; Goldenson et al. 2020; O’Connell et al. 2019; Phillips-Waller et al. 2021; Picavet et al. 2016; Rensch et al. 2021; Stiles et al. 2018; Voos et al. 2019; Yuki et al. 2017) and MST (Benowitz et al. 1988; Digard et al. 2013; Fant et al. 1999; Kotlyar et al. 2007; Lunell et al. 2020; Pickworth et al. 2014) indicate that the nicotine delivery and subjective effects of NPs tested are not likely to be greater than cigarette or MST. NPs may be potentially acceptable switching products for AS and adult MST users. A previous study demonstrated that nicotine PK and subjective responses are comparable across different flavor varieties for the 4 mg on!® NP; thus, findings from this study of mint-flavored on!® NPs extend to other on!® flavor varieties (Rensch et al. 2021).

The NPs, regardless of nicotine level, delivered nicotine far slower (higher *t*_max_ values) than participants’ OBCs and similar to OBMST. Additionally, the 1.5, 2, 3.5, and 4 mg NPs delivered lower peak nicotine concentrations (lower *C*_max_ values) than participants’ OBCs. The slower onset of nicotine delivery and lower peak concentration, along with the lower ratings of positive subjective effects relative to participants’ OBCs and/or OBMST, suggests likely lower reinforcing effects (Carter et al. 2009; Henningfield and Keenan 1993), and therefore, likely lower abuse potential for the NPs with nicotine levels of 1.5, 2, 3.5, and 4 mg. Our findings demonstrate that the NPs delivered nicotine in a manner consistent with their nicotine level (increased nicotine delivery with increasing nicotine level). These findings suggest that range of nicotine levels may allow individualized product use based on the specific needs of an AS or MST user.

**Fig. 3:**
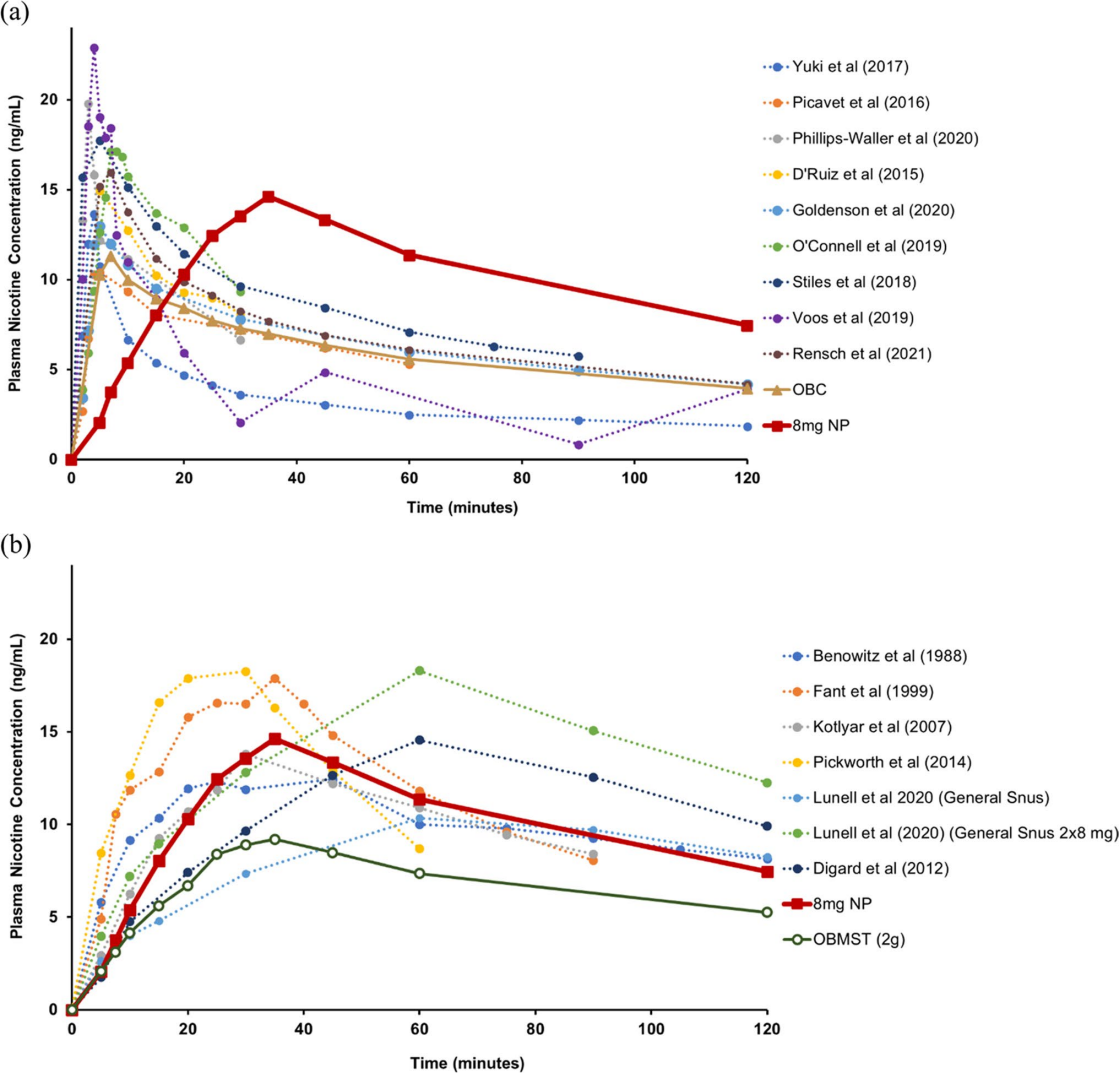
Plasma nicotine values over time during use of the 8 mg NP and representative published data for **a** cigarettes and **b** smokeless tobacco products. The plasma pharmacokinetic profiles from the published literature (dotted lines) are replotted from estimated values based on figures in the publications. For consistency, all data has been baseline adjusted. Results from this study (solid lines) are presented; only the 8 mg NP nicotine PK profile is presented because the 8 mg NP exhibited the highest nicotine PK relative to the lower nicotine level NPs. NP, nicotine pouch; OBC, own brand cigarette; OBMST, own brand moist smokeless tobacco

While we observed a higher *C*_max_ for the 8 mg NPs compared to OBC and OBMST used in this study, the mean nicotine *C*_max_ measured during use of the 8 mg NP (15.4 ng/mL) was within the range reported in published literature (Benowitz et al. 1988; D’Ruiz et al. 2015; Digard et al. 2013; Fant et al. 1999; Goldenson et al. 2020; Kotlyar et al. 2007; Lunell and Curvall 2011; Lunell et al. 2020; O’Connell et al. 2019; Phillips-Waller et al. 2021; Picavet et al. 2016; Pickworth et al. 2014; Stiles et al. 2018; Voos et al. 2019; Yuki et al. 2017) for cigarettes (11.8 to 23 ng/mL; Fig. 3a) and MST products (10.6 to 21.4 ng/ml; Fig. 3b). Also, in a previous study, we observed a higher mean *C*_max_ for OBCs was 17.7 ng/mL (Rensch et al. 2021), than that observed in this study (12.2 ng/mL). Similarly, the mean *C*_max_ values observed with OBMST (9.8 mg/mL) were lower than those reported in published literature (range of 12 to 19 ng/mL; Benowitz and Gourlay 1997; Benowitz et al. 1988; Fant et al. 1999; Kotlyar et al. 2007; Pickworth et al. 2014). Overall, based on the study results and literature reported PK values for cigarettes and MST, the abuse potential for the NPs is not likely to be higher than cigarettes or MST currently available in the US market.

Several factors could account for the lower-than-expected nicotine delivery from OBCs and OBMST in this study. While we have no direct evidence, the product use behavior may be different among exclusive smokers or exclusive MST users vs. dual users of cigarettes and MST products. There is some evidence of differential product use behavior; for example, Felicione et al. (2020) observed significantly lower cotinine levels among dual users on days when they only smoked cigarettes as opposed to when they dual used both cigarettes and MST. Additional factors likely contributing to the lower-than-expected nicotine delivery could be due to differences in own brand products or inherent inter-subject variability, including variations in use behavior, such as the size of the MST quid used in the ad libitum vs. controlled use periods. For example, the percent coefficient of variation was relatively higher for OBC (75.4%) in this study (Supplementary Table 2) compared to that reported for OBC (42.4%) by Rensch et al. (2021).

During the ad libitum use, participants reported that the NPs were satisfying, pleasant, and reduced craving a cigarette and urges to smoke (based on the QSU-Brief scores). However, the magnitude of change was relatively smaller compared to that observed with OBCs. Similar results were observed based on the responses to mCEQ related to satisfaction, psychological reward, enjoyment of sensation, and craving reduction.

Controlled use of the NPs resulted in a reduction in scores on similar items (e.g., urges to smoke, craving a cigarette, pleasantness, and satisfaction) addressed in the TNW and DEP Questionnaires. However, the magnitude of change was lower relative to participants’ OBCs and OBMST. The maximum change in cigarette craving and urges to smoke were not statistically significantly different for the 8 mg NP relative to OBC and OBMST (Supplementary Table 3). The maximum change in these subjective outcomes occurred later for 8 mg NP (15 min) than for OBC (5 min) and at a similar time to OBMST. These data suggest that the 8 mg NP can provide some cigarette craving relief, despite lower positive subjective ratings of the product’s effects. Nonetheless, the aversion factor score was highest for the 8 mg NP relative to OBC and OBMST (Supplementary Fig. 1).

The time-course of maximum reduction in nicotine withdrawal symptoms was similar among all NPs and OBMST and occurred 10 to 25 min later than with OBCs. Lower reinforcing efficacy can be expected from the NPs, regardless of nicotine level, because despite the positive subjective effects and some cigarette craving relief, the magnitude of change was lower than participants’ OBCs or OBMST. These observations suggest that, regardless of nicotine level, the NPs relieve nicotine withdrawal symptoms, but not to the same extent as smoking.

Potential limitations should be considered when drawing broad conclusions from this study. First, we did not directly measure dependence potential of the NPs because validated methods do not exist for such products. Additionally, even if such a measurement tool existed, it would be difficult to distinguish dependence resulting solely from the NPs because of the confounding effects of pre-existing nicotine dependence inherent to the study population since they are current tobacco users. Second, the study design involved measurement of subjective responses after a relatively brief duration of a 4-h period of in-clinic ad libitum product use. The lower subjective response results seen with the NPs compared to OBCs could possibly be due to NPs being novel and different, whereas the study participants are more familiar with their OBCs. Product familiarity may play an important role in the favorable subjective responses. Nonetheless, the in-clinic product use allowed for obtaining reliable observations since the product use took place in the presence of the clinic staff. Third, the controlled use condition of 30-min duration for a single pouch may not reflect typical real-world usage behavior. Vansickel et al. observed that typical use behavior for these pouches under actual use conditions is around 15 min (Vanickel et al. 2021); thus, the 30-min controlled use condition may reflect an extreme condition. Finally, the relatively small sample size and lack of sufficient gender balance may be a potential limitation as well. However, as described in the “Materials and methods” section, the sample size is reasonable and is typical of other studies examining PK and subjective effects across different tobacco/nicotine conditions (Carter et al. 2009; Cobb et al. 2010; Cox et al. 2001; Gray et al. 2008; Hatsukami et al. 2004; Kotlyar et al. 2007; Lunell and Curvall 2011; Perkins et al. 1997). Additionally, gender balance in this study reflects the population of ST product users, as it is well established that the vast majority of ST users are predominantly male. According to Centers for Disease Control and Prevention, non-Hispanic whites have the highest prevalence of ST use, and the number of women who use ST products is so small that statistically reliable estimates could not even be calculated (CDC 2021). Nonetheless, these limitations may restrict the generalizability to all tobacco/nicotine product users, particularly since differences in male and female nicotine PK have been reported by Benowitz et al. (2006). Despite these limitations, the nicotine PK analysis and the subjective measures used in this study are well-established methods for evaluating the abuse potential of tobacco/nicotine products (Carter et al. 2009; Cobb et al. 2010; Cox et al. 2001; Gray et al. 2008; Hanson et al. 2009; Henningfield and Keenan 1993; Lunell and Curvall 2011; Lunell et al. 2020; Vansickel et al. 2010, 2021a, b).

Overall, results from this study demonstrated that the 1.5, 2, 3.5, and 4 mg NPs likely have a lower abuse potential relative to OBCs and OBMST and the 8 mg NP is not likely to exhibit higher abuse potential than OBCs and OBMST. The abuse liability or dependence potential of a tobacco product must be considered in the context of that product’s harm reduction potential. Assessment of the abuse potential of the NPs informs the likelihood of switching from cigarettes and MST. In an actual use study, a modest proportion of AS and MST product users (who did not intend to quit using tobacco) switched completely to NPs when allowed open access to NPs under near real-world conditions over 6 weeks (Becker et al. 2021). In that actual use study, approximately 28% of AS and 72% of ST product users switched completely to NPs at the end of 6 weeks. These data demonstrate that the NPs are likely to be adopted by a portion of AS and MST users. Reduction in harm can only be achieved if adult tobacco users are willing to switch products. Therefore, for a tobacco/nicotine product to have the potential for reducing risk, the associated abuse potential should be sufficiently high enough, but not higher, than that of cigarettes (Institute of Medicine 2012). The abuse potential for the NPs, assessed in this study, is not likely to be higher than cigarettes or MST products and the NPs may be potentially acceptable substitutes for cigarettes.

## Supplementary Information

Below is the link to the electronic supplementary material.

Supplementary file1 (DOCX 84.9 KB)

## Declarations

This study was funded by Altria Client Services LLC, and all authors are employees of Altria Client Services LLC. The Advarra Institutional Review Board (IRB) (Columbia, MD) reviewed and approved this study. The study was conducted in accordance with Food and Drug Administration (FDA) regulations as described in the Code of Federal Regulations (CFR) 21 Parts 50 and 56, Department of Health and Human Services regulations as described in 45 CFR 46, guidelines resulting from the International Council for Harmonisation (ICH) E6 Good Clinical Practice (GCP), the European Union (EU) directive 2001/20/EC, and the ethical principles set forth in the Declaration of Helsinki. In addition, the IRB operates in compliance with the portions of the Health Insurance Portability and Accountability Act of 1996 (HIPAA Privacy Rule) that apply to research, as described in 45 CFR Parts 160 and 164. All participants in this study reviewed, signed, and dated the informed consent form prior to study initiation.
