# Supplementary material for: Nicotine pharmacokinetics and subjective responses after using nicotine pouches with different nicotine levels compared to combustible cigarettes and moist smokeless tobacco in adult tobacco users
Source: Psychopharmacology (Berl). 2022 Jul 23;239(9):2863–73. doi: 10.1007/s00213-022-06172-y (PMC9385814; doi:10.1007/s00213-022-06172-y)
Supplement: Supplementary file 1 — Supplementary file1 (DOCX 84.9 KB) [file 213_2022_6172_MOESM1_ESM.docx]

**Supplementary Material**

**Article Title:** Nicotine pharmacokinetics and subjective response among adult tobacco users using nicotine pouches with different nicotine levels compared to combustible cigarettes and moist smokeless tobacco products

**Journal name:** *Psychopharmacology*

**Authors:** Jianmin Liu, Jesse Rensch, Jingzhu Wang, Xiaohong Jin, Andrea Vansickel, Jeffery Edmiston, Mohamadi Sarkar

Altria Client Services, Richmond

**Corresponding Author:**

Mohamadi Sarkar, M.Pharm., Ph.D., FCP

Altria Client Services LLC

mohamadi.a.sarkar@altria.com

# Supplementary Table 1. Subjective Measures

| **Assessment Name** | **When**  **Administered** | **Items** |
| --- | --- | --- |
| Questionnaire on  Smoking Urges  (QSU-Brief) | Within 5 minutes before and ~1 minute after the 4-hour *ad libitum* use period | *I have a desire for a cigarette right now.*  *Nothing would be better than smoking a cigarette right now.*  *If it were possible, I would probably smoke right now.*  *I could control things better right now if I could smoke.*  *All I want right now is a cigarette.*  *I have an urge for a cigarette.*  *A cigarette would taste good right now.*  *I would do almost anything for a cigarette right now.*  *Smoking would make me less depressed.*  *I am going to smoke as soon as possible.*    Participants responded to each item using a 7-point scale: *Strongly Disagree* (1) through *Strongly Agree* (7).  Yields two factor scores: Factor 1 (Desire and Intention to Smoke) and Factor 2 (Anticipation of Relief from Negative Affect). |

| Modified  Cigarette  Evaluation  Questionnaire  (mCEQ) | Immediately after the 4-hour *ad libitum* use period | **Nicotine pouches**  *Was using the pouches satisfying?*  *Did the pouches taste good?*  *Did you enjoy the sensations in your mouth?*  *Did using the pouches calm you down?*  *Did using the pouches make you feel more awake?*  *Did using the pouches make you feel less irritable?*  *Did using the pouches help you concentrate?*  *Did using the pouches reduce your hunger for food?*  *Did using the pouches make you dizzy?*  *Did using the pouches make you nauseous?*  *Did using the pouches immediately relieve your craving for a cigarette? Did you enjoy using the pouches?*  **Cigarettes**  *Was smoking cigarettes satisfying?*  *Did the cigarettes taste good?*  *Did you enjoy the sensation in your throat and chest?*  *Did smoking cigarettes calm you down?*  *Did smoking cigarettes make you feel more awake?*  *Did smoking cigarettes make you feel less irritable?*  *Did smoking cigarettes help you concentrate?*  *Did smoking cigarettes reduce your hunger for food?*  *Did smoking cigarettes make you dizzy?*  *Did smoking cigarettes make you nauseous?*  *Did smoking cigarettes immediately relive your craving for a cigarette? Did you enjoy smoking cigarettes?*  **Moist Smokeless Tobacco (MST)** *Was using the MST product satisfying?*  *Did the MST product taste good?*  *Did you enjoy the sensation in your mouth?*  *Did using the MST product calm you down?*  *Did using the MST product make you feel more awake?*  *Did using the MST product make you feel less irritable?*  *Did using the MST product help you concentrate?*  *Did using the MST product reduce your hunger for food?*  *Did using the MST product make you nauseous?*  *Did using the MST product immediately relieve your craving for a cigarette?*  *Did you enjoy using the MST product?*    Participants rated each item on a 7-point scale: 1-*not at all*, 2-*very little*, 3-*a little*, 4-*moderately*, 5-*a lot*, 6-*quite a lot*, 7-*extremely*. Yields five factor scores: Satisfaction, Enjoyment of Sensations, Psychological Reward, Craving, and Aversion. |
| --- | --- | --- |
| **Assessment Name** | **When**  **Administered** | **Items** |
| Tobacco/Nicotine  Withdrawal  (TNW)  Questionnaire | 5 minutes before and  immediately after the scheduled blood draws at  5, 15, 30, and 60 minutes during the controlled use period | *These phrases may or may not describe how you feel right now. Please respond to each word or phrase with how you feel RIGHT NOW...*  *Urges to smoke*  *Craving a Cigarette*    Participants responded to each item using a 100 mm visual analog scale anchored by *not at all* (0) and *extremely* (100). |
| Direct Effects of  Product (DEP)  Questionnaire | Immediately after the scheduled blood draws at  5, 15, 30, and 60 minutes during the controlled use period | *Is the product “Pleasant” right now?*  *Is the product “Satisfying” right now?*  *Is the product making you feel “Calm” right now?*  *Is the product helping you “Concentrate” right now?*  *Is the product making you feel more “Awake” right now?*  *Is the product making you feel “Sick” right now?*  *Is the product reducing your “Hunger” for food right now? Would you like “More” of the product right now?*    Participants responded to each item using a 100 mm visual analog scale anchored by *not at all* (0) and *extremely* (100). |
| Use the Product Again | Within 2 minutes after the 180-minute blood draw during the controlled use period | *If given the opportunity, I would want to use this product again*    Participants responded using a 100 mm bipolar visual analog scale anchored with *Definitely would* (50) and *Definitely would not* (-50) with *Don’t care* (0) as the neutral point.  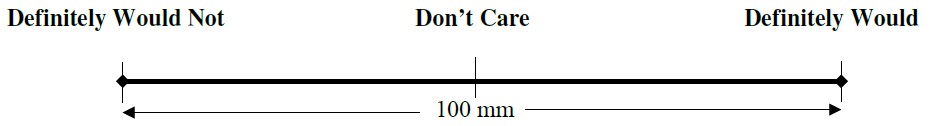 |

Supplementary Table 2. Summary of Baseline-adjusted Plasma Nicotine Pharmacokinetic Parameters

| **Product** |  | **AUC,**  **ng*min/mL** | **C_max,_ ng/mL** | **tmax,**  **min** | **kel, min^-1^** | **t_½_, min** |
| --- | --- | --- | --- | --- | --- | --- |
| 1.5 mg NP | n | 29 | 30 | 30 | 24 | 24 |
|  | Mean (SD) | 333.6 (130.29) | 3.5 (1.20) | 33.5  (7.00) | 0.005837  (0.0010743) | 122.6  (22.05) |
|  | G.mean/G.CV% | 307.0/ 44.9 | 3.256/ 38.4 | — | — | — |
| 2 mg NP | n | 29 | 29 | 29 | 24 | 24 |
|  | Mean (SD) | 462.0 (194.19) | 4.9 (2.11) | 32.5  (7.35) | 0.005936  (0.0012622) | 121.7  (24.68) |
|  | G.mean/G.CV% | 428.1/40.6 | 4.60/37.4 | — | — | — |
| 3.5 mg NP | n | 28 | 28 | 28 | 21 | 21 |
|  | Mean (SD) | 761.5 (290.66) | 7.5 (2.28) | 33.0  (5.42) | 0.006385  (0.0012548) | 113.2  (25.16) |
|  | G.mean/G.CV% | 706.0/42.5 | 7.10/35.3 | — | — | — |
| 4 mg NP | n | 28 | 28 | 28 | 24 | 24 |
|  | Mean (SD) | 871.9 (348.20) | 9.1 (3.48) | 33.8  (5.58) | 0.006428  (0.0011588) | 111.3  (20.29) |
|  | G.mean/G.CV% | 809.0/41.4 | 8.52/39.5 | — | — | — |
| 8 mg NP | n | 27 | 28 | 28 | 23 | 23 |
|  | Mean (SD) | 1554 (611.30) | 15.4 (5.51) | 33.9  (5.53) | 0.006624  (0.0011572) | 107.9  (19.8) |
|  | G.mean/G.CV% | 1449/39.2 | 14.50/37.2 | — | — | — |
| Participant's  OBC | n | 29 | 29 | 29 | 29 | 29 |
|  | Mean (SD) | 926.9 (402.28) | 12.2 (5.50) | 8.5  (3.60) | 0.005977  (0.0013108) | 121.1  (25.94) |
|  | G.mean/G.CV% | 808.4/71.7 | 10.52/75.4 | — | — | — |
| Participant's  OBMST | n | 29 | 29 | 29 | 24 | 24 |
|  | Mean (SD) | 1065 (410.30) | 9.8 (3.40) | 34.4  (6.64) | 0.006068  (0.0011985) | 118.9  (25.62) |
|  | G.mean/G.CV% | 994.7/38.5 | 9.230/37.0 | — | — | — |

In addition to arithmetic mean, G.mean and G.CV% were calculated for C_max_ and AUC using log-transformed data. AUC = area under the nicotine concentration-time curve from time 0 to 180 minutes; C_max_ = maximum measured plasma concentration; G.CV% = geometric coefficient of variation percentage; G.mean = geometric mean; kel = apparent first-order terminal elimination rate constant; NP = nicotine pouch; OBC = own brand cigarette; OBMST = own brand moist smokeless tobacco; SD = standard deviation; t_max_ = time to maximum measured plasma nicotine concentration; t_½_ = apparent first-order elimination half-life.

# Supplementary Table 3. Relative Comparisons Between Subjective Ratings (E_max_) of the Nicotine Pouches and Cigarettes

|  | **1.5 mg NP** | **2 mg NP** | **3.5 mg NP** | **4 mg NP** | **8 mg NP** | **OBC** | **OBMST** |
| --- | --- | --- | --- | --- | --- | --- | --- |
| **Tobacco/Nicotine Withdrawal Questionnaire** | | | | | | | |
| Urges to Smoke | 17.37^a,b^ | 22.48 | 22.48 | 18.23^a,b^ | 26.60 | 29.84 | 29.02 |
| Craving a Cigarette | 19.03^a,b^ | 19.80^a,b^ | 19.06^a,b^ | 20.93^a,b^ | 27.94 | 31.08 | 32.08 |
| **Direct Effects of Product Questionnaire** | | | | | | | |
| Is the product “Pleasant” right now | 48.37^a,b^ | 54.74^a,b^ | 53.46^a,b^ | 52.80^a,b^ | 51.98^a,b^ | 69.80 | 65.94 |
| Is the product “Satisfying” right  now | 46.29^a,b^ | 54.24^a,b^ | 53.31^a,b^ | 51.01^a,b^ | 51.42^a,b^ | 71.30 | 67.34 |
| Is the product making you feel  “Calm” right now | 42.47^a,b^ | 50.44^a,b^ | 49.16^a,b^ | 44.43^a,b^ | 48.74^a,b^ | 65.01 | 63.21 |
| Is the product helping you  “Concentrate” right now | 35.42^a,b^ | 45.00 | 43.04^a^ | 39.69^a,b^ | 40.41^a,b^ | 51.11 | 50.01 |
| Is the product making you feel more “Awake” right now | 40.78^a,b^ | 45.55 | 45.73 | 39.06^a,b^ | 42.30^b^ | 50.94 | 51.96 |
| Is the product making you feel “Sick” right now? | 16.70 | 13.59 | 15.64 | 18.54 | 26.75 | 20.78 | 19.86 |
| Is the product reducing your  “Hunger” for food right now? | 26.09^a,b^ | 28.58^a,b^ | 28.79^a^ | 30.50 | 34.17 | 38.90 | 37.24 |
| Would you like “More” of the product right now? | 65.48 | 66.34 | 63.30^a^ | 62.31^a^ | 51.27^a,b^ | 73.98 | 68.38 |

The Tobacco/Nicotine Withdrawal (TNW) Questionnaire, the Direct Effects of Product (DEP) Questionnaire, and the Use the Product Again bipolar visual analog scale are described in Supplementary Table 1. The comparison results were based on linear mixed model for analysis of variance. The mixed model includes product sequence, period, and product as fixed effects and subject nested within product sequence as a random effect. Mixed model with a default (variance component) covariance structure was used. Parameters were natural log-transformed prior to analysis. Data are presented as least-squares means.

^a^ Statistically significantly different as compared to OBC (*p* < 0.05). ^b^ Statistically significantly different as compared to OBMST (*p* < 0.05).

NP = nicotine pouch; OBC = own brand cigarette; OBMST = own brand moist smokeless tobacco.

# Supplementary Fig. 1 Modified Cigarette Evaluation Questionnaire Factor Scores Following 4-hour *Ad Libitum* Use

1.5 mg NP

**M**

**e**

**a**

**n**

**(**

**S**

**E**

**)**

**F**

**a**

**c**

**t**

**o**

**r**

**S**

**c**

**o**

**r**

**e**

**1**

**2**

**3**

**4**

**5**

**6**

**7**

2 mg NP

3.5 mg NP

4 mg NP

8 mg NP

OBC

OBMST

**Satisfaction Psychological Aversion Enjoyment Craving**

## Reward of Sensation Reduction

Questions included in the Modified Cigarette Evaluation Questionnaire are described in Supplementary Table 1. NP = nicotine pouch; OBC = own brand cigarette; OBMST = own brand moist smokeless tobacco; SE = standard error.

# Supplementary Fig. 2 Percentage of Participants Responding Within Each Response Region of the Bipolar VAS for Want to Use the Product Again

**1**

**.**

**5**

**m**

**g**

**N**

**P**

**2**

**m**

**g**

**N**

**P**

**3**

**.**

**5**

**m**

**g**

**N**

**P**

**4**

**m**

**g**

**N**

**P**

**8**

**m**

**g**

**N**

**P**

**O**

**B**

**C**

**O**

**B**

**M**

**S**

**T**

**0**

**50**

**100**

**P**

**e**

**r**

**c**

**e**

**n**

**t**

**a**

**g**

**e**

**o**

**f**

**P**

**a**

**r**

**t**

**i**

**c**

**i**

**p**

**a**

**n**

**t**

**s**

**R**

**e**

**s**

**p**

**o**

**n**

**d**

**i**

**n**

**g**

**W**

**i**

**t**

**h**

**i**

**n**

**E**

**a**

**c**

**h**

**R**

**e**

**g**

**i**

**o**

**n**

**o**

**f**

**t**

**h**

**e**

**B**

**i**

**p**

**o**

**l**

**a**

**r**

**V**

**A**

**S**

Negative (-50 to <0)

Neutral (0)

Positive (>0 to 50)

**66**

**%**

**55**

**%**

**%**

**61**

**61**

**%**

**46**

**%**

**77**

**%**

**93**

**%**

**3**

**%**

**10**

**%**

**%**

**11**

**7**

**%**

**18**

**%**

**4**

**%**

**%**

**31**

**34**

**%**

**29**

**%**

**32**

**%**

**36**

**%**

**19**

**%**

**7**

**%**

**(20.5)**

**(23.1)**

**(24.3)**

**(25.9)**

**(21.6)**

**(37.2)**

**(29.7)**

**(-25.9)**

**(-27.9)**

**(-26.1)**

**(-20.9)**

**(-36.3)**

**(-11.4)**

**(-29.0)**

Each bar represents the percentage of participants, rounded to the nearest whole number, who responded within the negative, neutral, or positive response regions of the bipolar VAS by product on the Use the Product Again Questionnaire (described in Supplementary Table 1). Average scores within the positive and negative response regions are depicted parenthetically.

NP = nicotine pouch; OBC = own brand cigarette; OBMST = own brand moist smokeless tobacco; VAS = visual analog scale.
